# The CRAC channel inhibitor Auxora reprograms pathogenic alveolar macrophage–T cell circuits in viral pneumonia

**DOI:** 10.1172/JCI209675

**Published:** 2026-07-28

**Authors:** S. Marina Casalino-Matsuda, Vijeeth Guggilla, Catherine A. Gao, Kaitlyn E. DeMeulenaere, Luisa Cusick, Samuel W. Fenske, Zhan Yu, Ziyan Lu, Suchitra Swaminathan, Rogan A. Grant, Maxwell J. Schleck, Murali Prakriya, Sudarshan Hebbar, Kenneth Stauderman, Helen K. Donnelly, Chiagozie I. Pickens, Luisa Morales-Nebreda, Richard G. Wunderink, Alexander V. Misharin, Benjamin D. Singer, G.R. Scott Budinger

**Affiliations:** 1 Division of Pulmonary and Critical Care Medicine, Department of Medicine,; 2 Health Sciences Integrated Program, Health and Biomedical Informatics Track, and; 3 Department of Pharmacology, Northwestern University Feinberg School of Medicine, Chicago, Illinois, USA.; 4 CalciMedica Inc., La Jolla, California, USA.; 5 Jesse Brown VA Medical Center, Chicago, Illinois, USA.; 6 NU SCRIPT Study Investigators (detailed in Supplemental Acknowledgments).

**Keywords:** Immunology, Infectious disease, Pulmonology, Calcium channels, Macrophages, T cells

## Abstract

The CRAC channel inhibitor Auxora Reprograms Pathogenic Alveolar Macrophage–T Cell Circuits in Viral Pneumonia

**To the Editor:** Severe SARS-CoV-2 pneumonia is perpetuated by inflammatory circuits between activated T cells and monocyte-derived alveolar macrophages (MoAMs) (1–4). T cells and macrophages express ORAI1 and STIM1, which form calcium release–activated calcium (CRAC) channels that allow extracellular calcium entry in response to ER calcium store depletion (5). In a randomized, placebo-controlled, multicenter phase II trial (CARDEA), Auxora, a CRAC channel inhibitor, reduced all-cause 30-day mortality by 56% in patients with severe SARS-CoV-2 pneumonia (6).

Concurrent with the CARDEA trial, we conducted an exploratory mechanistic study in a small cohort of critically ill, mechanically ventilated patients with laboratory-confirmed SARS-CoV-2 pneumonia (NCT04661540) (Figure 1A, Supplemental Figure 1A, and Supplemental Table 1; supplemental material available online with this article; https://doi.org/10.1172/JCI209675DS1). The study was not powered to detect differences in mortality, duration of ventilation (Figure 1B), or SARS-CoV-2 viral titers (Supplemental Figure 1B), which did not differ in patients receiving Auxora or placebo. Plasma levels of Auxora were 1,959 ± 755.4 nM, 30 minutes after the first infusion, and 2,065 ± 586.0 nM, 30 minutes after the third infusion, and were undetectable 24 hours or more later (Supplemental Figure 1C). We measured plasma levels of 72 cytokines (2) and found that CCL8 (aka MIP-2), which is mostly produced by macrophages and involved in the recruitment of monocytes, and PDGF-AA, a mitogen and chemoattractant of mesenchymal cells, were substantially reduced after Auxora, but not placebo, treatment (Figure 1C).

Auxora treatment did not result in differences in bronchoalveolar lavage (BAL) fluid immune cell composition, as measured by flow cytometry (Supplemental Figure 1D) or single-cell RNA-seq (scRNA-seq) of clustered and annotated cells as previously described (1) (Supplemental Figure 1, E–G). We did not detect differences in gene expression in any cell population as a function of Auxora treatment (Supplemental Figure 1, H and I). *CCL8* was lower in MoAMs from patients treated with Auxora compared with pretreatment baseline levels (Supplemental Figure 1I), but the differences did not reach statistical significance. SARS-CoV-2 transcripts were detected in MoAMs at baseline and were infrequently detected following Auxora treatment. Statistical differential expression testing was not performed for viral genes because of sparse viral transcript counts (Supplemental Figure 1I).

We also compared the pharmacodynamic responses of T cells and macrophages to Auxora using PBMCs from healthy volunteers after differentiating monocytes into monocyte-derived macrophages (MDMs). In T cells, we found that Auxora blocked Ca^2+^ entry in a dose-dependent fashion, beginning at a concentration of 50 nM (Supplemental Figure 2, A–C). In contrast, Auxora inhibited CRAC channel–mediated Ca^2+^ entry in MDMs at a concentration of 500 nM (Supplemental Figure 2, D–F).

To test whether Auxora inhibits inflammatory circuits between MDMs and T cells, we cultured PBMCs from healthy volunteers for 24 hours and then treated them with Auxora (500 nM) or vehicle, followed 2 hours later by treatment with anti-CD3/CD28 antibodies or IFN-γ, and processed them for scRNA-seq 24 hours later (Figure 1D and Supplemental Figure 2, G–J). Auxora did not induce cytotoxicity at this concentration (Supplemental Figure 2G). Principal component analysis (PCA) revealed that treatment with anti-CD3/CD28 antibodies induced transcriptional reprogramming in CD4^+^ and CD8^+^ T cells and MDMs that was attenuated by Auxora (Figure 1, E, G, and I). We identified large numbers of differentially expressed genes (DEGs) in CD4^+^ and CD8^+^ T cells and MDMs treated anti-CD3/CD28 antibodies, a majority of which were affected by Auxora (Supplemental Figure 3A). Genes attenuated by Auxora included those encoding secreted cytokines involved in T cell activation, for example *IL2*, *TNF*, and *IFNG* (Figure 1, F, H, and J). Gene set enrichment analysis (GSEA) revealed activation of several common inflammatory pathways in T cells and MDMs that were inhibited by Auxora — for example, TNF-α signaling via NF-κB and IL-2/STAT5 signaling (Supplemental Figure 3, B and C).

As MDMs do not express CD3 or CD28, their activation in response to anti-CD3/CD28 antibodies is likely attributable to indirect signals from T cells (Figure 1J; Supplemental Figure 2, K and L; and Supplemental Figure 3F). Therefore, we examined the effect of Auxora on the response of T cells and MDMs to recombinant IFN-γ, the primary cytokine released by T cells in the scRNA-seq data (Supplemental Figure 3, E and F). Only a small fraction of the genes induced in T cells by anti-CD3/CD28 antibodies were induced by IFN-γ. In contrast, genes induced in MDMs treated with IFN-γ substantially overlapped with those induced by anti-CD3/CD28 antibodies and IFN-γ in MDMs (Supplemental Figure 2, K and L, and Supplemental Figure 3F). These exploratory results suggest that Auxora reprograms T cell–MoAM circuits, mediated in large part by IFN-γ. Identifying pneumonia endotypes characterized by similar circuits might guide the selection of participants for future clinical trials of Auxora in patients with pneumonia.

Further information can be found in Supplemental Methods and Supplemental Data Set 1.

## Conflict of interest

BDS holds a US patent (US patent no. 10,905,706 B2; “Compositions and Methods to Accelerate Resolution of Acute Lung Inflammation”) and serves on the scientific advisory board of Zoe Biosciences. AVM received research grants from AbbVie and Merck and consulting fees from Boehringer Ingelheim. SH and KS are employees of CalciMedica and own shares in CalciMedica. RGW serves as a consultant for CalciMedica.

## Funding support

This work is the result of NIH funding, in whole or in part, and is subject to the NIH Public Access Policy. Through acceptance of this federal funding, the NIH has been given a right to make the work publicly available in PubMed Central.

- National Heart, Lung, and Blood Institute (NHLBI), NIH grant K23HL169815 (to CAG).
- Parker B. Francis Opportunity Award (to CAG).
- American Thoracic Society Unrestricted Grant (to CAG).
- National Institute of Allergy and Infectious Diseases (NIAID), NIH grants U19 AI135964 and R01 AI158530 (to RGW).
- NHLBI, NIH grants R01 HL149883, P01 HL154998, and U01TR003528 (to RGW).
- NIH grants R01HL149883, R01HL153122, P01HL154998, P01AG049665, U19AI135964, and U19AI181102 (to BDS).
- NIH grants U19AI135964, P01AG049665, R01HL147575, P01HL071643, U54AG079754, R01HL145478, R01HL147290, R01HL173940, U19AI181102, and R01HL154686 (to GRSB).
- US Department of Veterans Affairs grant I01CX001777 (to GRSB).
- Simpson Querrey Lung Institute for Translational Sciences (to GRSB).
- NIH grants U19AI135964, P01AG049665, P01HL154998, P01HL169188, U19AI181102, R01HL153312, R01HL158139, and R01ES034350 (to AVM).
- AbbVie and Merck research grants (to AVM).

## Supplementary Material

Supplemental data

Supplemental data set 1

Supporting data values

## Figures and Tables

**Figure 1:**
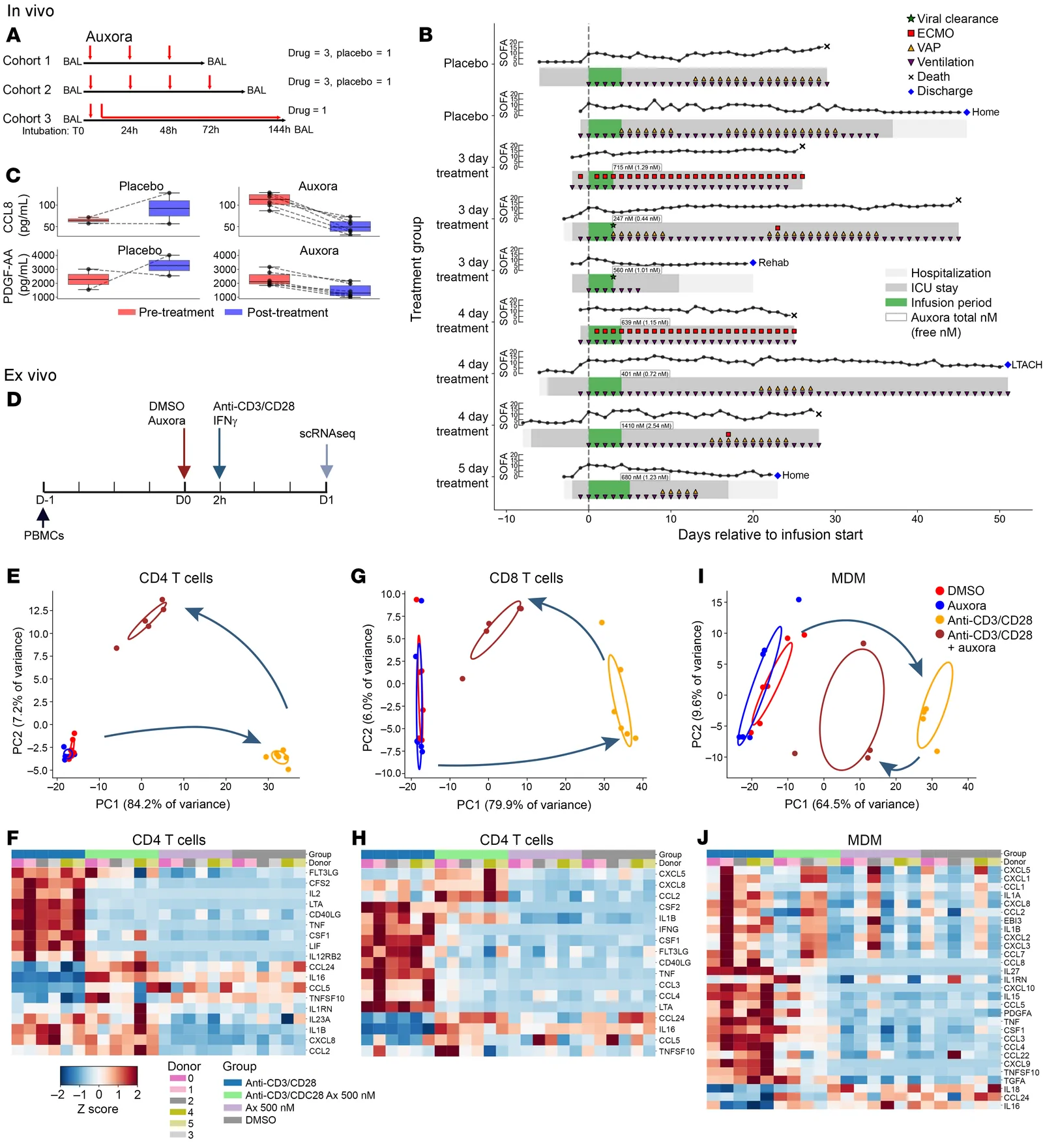
Auxora targets T cells to inhibit T cell–macrophage circuits during SARS-CoV-2 pneumonia. (**A**) Schematic illustrating protocol-driven interventions. (**B**) Swimmer plots of the ICU course in patients enrolled in the trial. SOFA, sequential organ failure assessment (score assessing viral clearance); ECMO, extracorporeal membrane oxygenation initiated; VAP, ventilator-associated pneumonia episode. (**C**) Plasma CCL8 and PDGF-AA levels before and after placebo or Auxora infusion. Significance was determined by linear mixed models using a paired design. (**D**) Schematic for experiments in PBMCs from 5 different blood donors in **E**–**J**. (**E**–**J**) Genes were identified as differentially expressed by a negative binomial generalized linear model using an ANOVA-like test for differential expression between any conditions in the dataset (FDR *q* < 0.05). Ax, Auxora. (**E**) PCA of DEGs in CD4^+^ T cells. (**G**) CD8^+^ T cells (**I**) MDMs. Heatmaps demonstrating the expression of selected genes of interest among the DEGs in (**F**) CD4^+^ T cells, (**H**) CD8^+^ T cells, and (**J**) MDMs.

## Version 1 07/28/2026

In-Press Preview

## Version 2 09/15/2026

Electronic publication
