# Supplementary material for: The CRAC channel inhibitor Auxora reprograms pathogenic alveolar macrophage–T cell circuits in viral pneumonia
Source: J Clin Invest. 2026 Jul 28;136(18):e209675. doi: 10.1172/JCI209675 (PMC13574160; doi:10.1172/JCI209675)
Supplement: Supplementary file 1 [file jci-136-209675-s142.pdf]

The CRAC channel inhibitor Auxora Reprograms Pathogenic Alveolar Macrophage–T Cell Circuits in Viral Pneumonia

S. Marina Casalino Matsuda[1*], Vijeeth Guggilla[2*], Catherine A. Gao[1], Kaitlyn E. Demeulenaere[3], Luisa Cusick[1], Samuel W. Fenske[1], Zhan Yu[1], Ziyan Lu[1], Suchitra Swaminathan[1], Rogan A. Grant[1], Maxwell J. Schleck[1], Murali Prakriya[3], Sudarshan Hebbar[4], Kenneth Stauderman[4], Helen K. Donnelly[1], Chiagozie I. Pickens[1], Luisa Morales-Nebreda[1], The NU SCRIPT Study Investigators, Richard G. Wunderink[1#], Alexander V. Misharin[1#], Benjamin D. Singer[1#], G.R. Scott Budinger[1,5#]

[1] Division of Pulmonary and Critical Care Medicine, Department of Medicine, Northwestern University Feinberg School of Medicine, Chicago, IL, USA

[2] Health Sciences Integrated Program, Health and Biomedical Informatics Track, Northwestern University Feinberg School of Medicine, Chicago, IL, USA

[3] Department of Pharmacology, Northwestern University Feinberg School of Medicine, Chicago, IL, USA.

[4] CalciMedica, Inc, La Jolla, CA, USA

[5] Jesse Brown VA Medical Center, Chicago, IL, USA

*SMCM and VG contributed equally to this work

#RGW, AVM, BDS, and GRSB contributed equally to this work

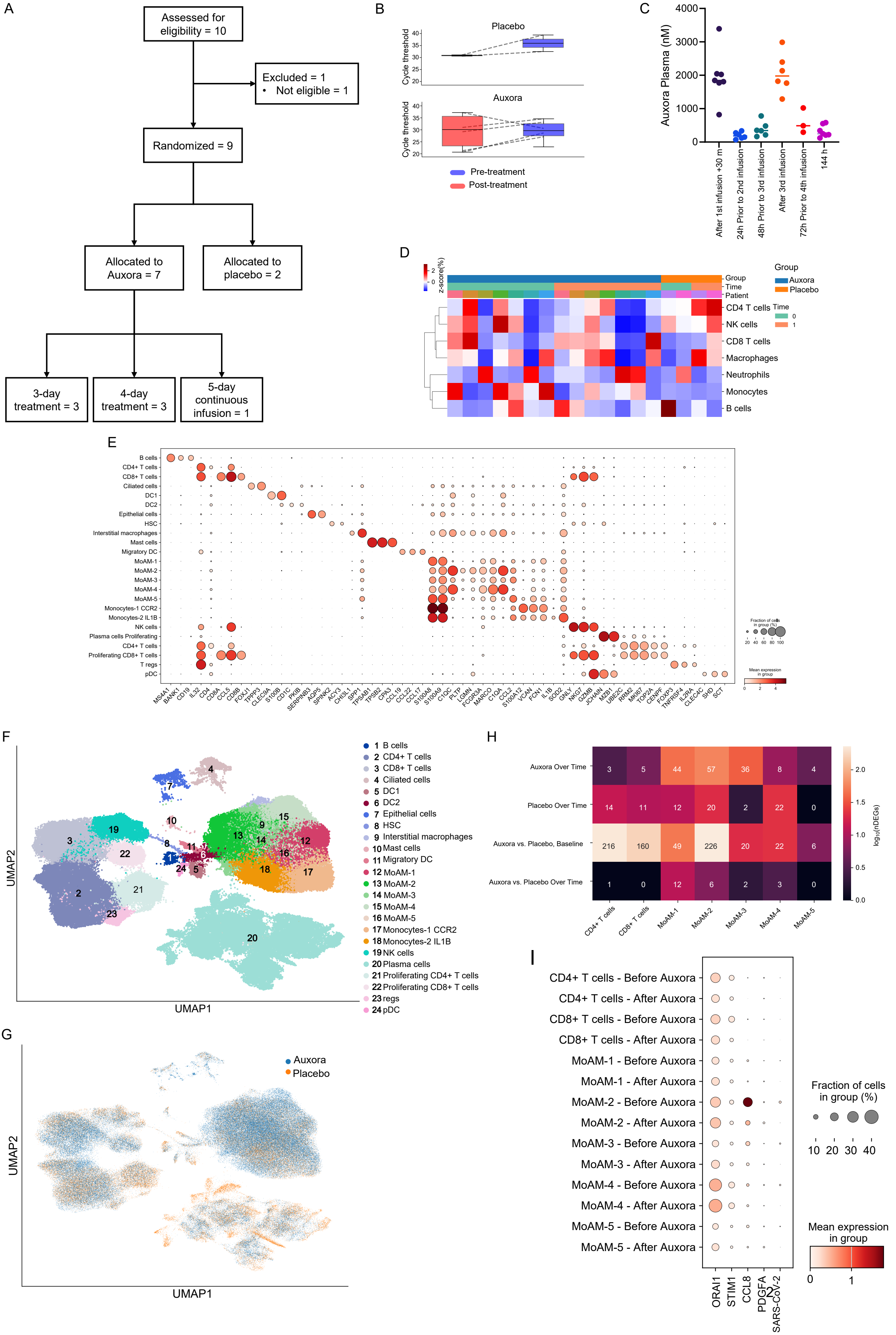
A
Assessed for eligibility = 10
Excluded = 1
• Not eligible = 1
Randomized = 9
Allocated to Auxora = 7
Allocated to placebo = 2
3-day treatment = 3
4-day treatment = 3
5-day continuous infusion = 1
B
Placebo
Auxora
Cycle threshold
Pre-treatment
Post-treatment
C
Auxora Plasma (nM)
After 1st infusion +30 m
24h Prior to 2nd infusion
48h Prior to 3rd infusion
After 3rd infusion
72h Prior to 4th infusion
144 h
D
z-score(%)
Group
Time
Patient
Auxora
Placebo
CD4 T cells
NK cells
CD8 T cells
Macrophages
Neutrophils
Monocytes
B cells
E
B cells
CD4+ T cells
CD8+ T cells
Ciliated cells
DC1
DC2
Epithelial cells
HSC
Interstitial macrophages
Mast cells
Migratory DC
MoAM-1
MoAM-2
MoAM-3
MoAM-4
MoAM-5
Monocytes-1 CCR2
Monocytes-2 IL1B
NK cells
Plasma cells Proliferating
CD4+ T cells
Proliferating CD8+ T cells
T regs
pDC
Fraction of cells in group (%)
Mean expression in group
F
UMAP1
UMAP2
1 B cells
2 CD4+ T cells
3 CD8+ T cells
4 Ciliated cells
5 DC1
6 DC2
7 Epithelial cells
8 HSC
9 Interstitial macrophages
10 Mast cells
11 Migratory DC
12 MoAM-1
13 MoAM-2
14 MoAM-3
15 MoAM-4
16 MoAM-5
17 Monocytes-1 CCR2
18 Monocytes-2 IL1B
19 NK cells
20 Plasma cells
21 Proliferating CD4+ T cells
22 Proliferating CD8+ T cells
23 regs
24 pDC
G
Auxora
Placebo
UMAP1
UMAP2
H
Auxora Over Time
Placebo Over Time
Auxora vs. Placebo, Baseline
Auxora vs. Placebo Over Time
CD4+ T cells
CD8+ T cells
MoAM-1
MoAM-2
MoAM-3
MoAM-4
MoAM-5
log10(nDEGs)
I
CD4+ T cells - Before Auxora
CD4+ T cells - After Auxora
CD8+ T cells - Before Auxora
CD8+ T cells - After Auxora
MoAM-1 - Before Auxora
MoAM-1 - After Auxora
MoAM-2 - Before Auxora
MoAM-2 - After Auxora
MoAM-3 - Before Auxora
MoAM-3 - After Auxora
MoAM-4 - Before Auxora
MoAM-4 - After Auxora
MoAM-5 - Before Auxora
MoAM-5 - After Auxora
ORAI1
STIM1
CCL8
PDGFA
SARS-CoV-2
Fraction of cells in group (%)
Mean expression in group

__Supplemental Figure 1__: **Auxora targets T cells to inhibit T cell-macrophage circuits during SARS-CoV-2 pneumonia**. **A**, CONSORT diagram of the study. **B**, Viral load before and after placebo or Auxora infusion estimated by PCR cycle threshold in BAL fluid samples. **C**, Auxora total concentrations in plasma. **D**, Cell abundance measured by flow cytometry analysis of BAL samples. Samples were clustered by Manhattan distance using complete linkage. **E,** Dot plot illustrating expression of marker genes for scRNA-seq cluster annotations. Cell phenotypes are listed on the y-axis and genes (features) are listed along the x-axis. Dot size reflects the percentage of cells in a cluster expressing each gene; dot color reflects expression level. **F,** Uniform manifold approximation and projection (UMAP) plot showing integrated analysis of BAL cells from patients with severe SARS (n = 9). **G**, UMAP plot illustrating integrated analysis of BAL cells isolated from patients with severe SARS-CoV-2 pneumonia (n=9) and labeled for placebo or Auxora treatments. **H**, cell abundances before and after administration of Auxora measured using scRNA-sequencing. Neutrophils were excluded by our cryopreservation procedure and their abundance was estimated from flow cytometry data. **I**, Dot plot of indicated ORAI1, STIM1, CCL8, and PDGFA transcripts, and the sum of SARS-CoV-2 transcripts, in different cell types before and after Auxora treatment. Differences were not significant based on differential gene expression analysis using DESeq2 ($q > 0.05$, Wald test with FDR correction). Statistical testing not performed for SARS-CoV-2 genes due to sparse expression.

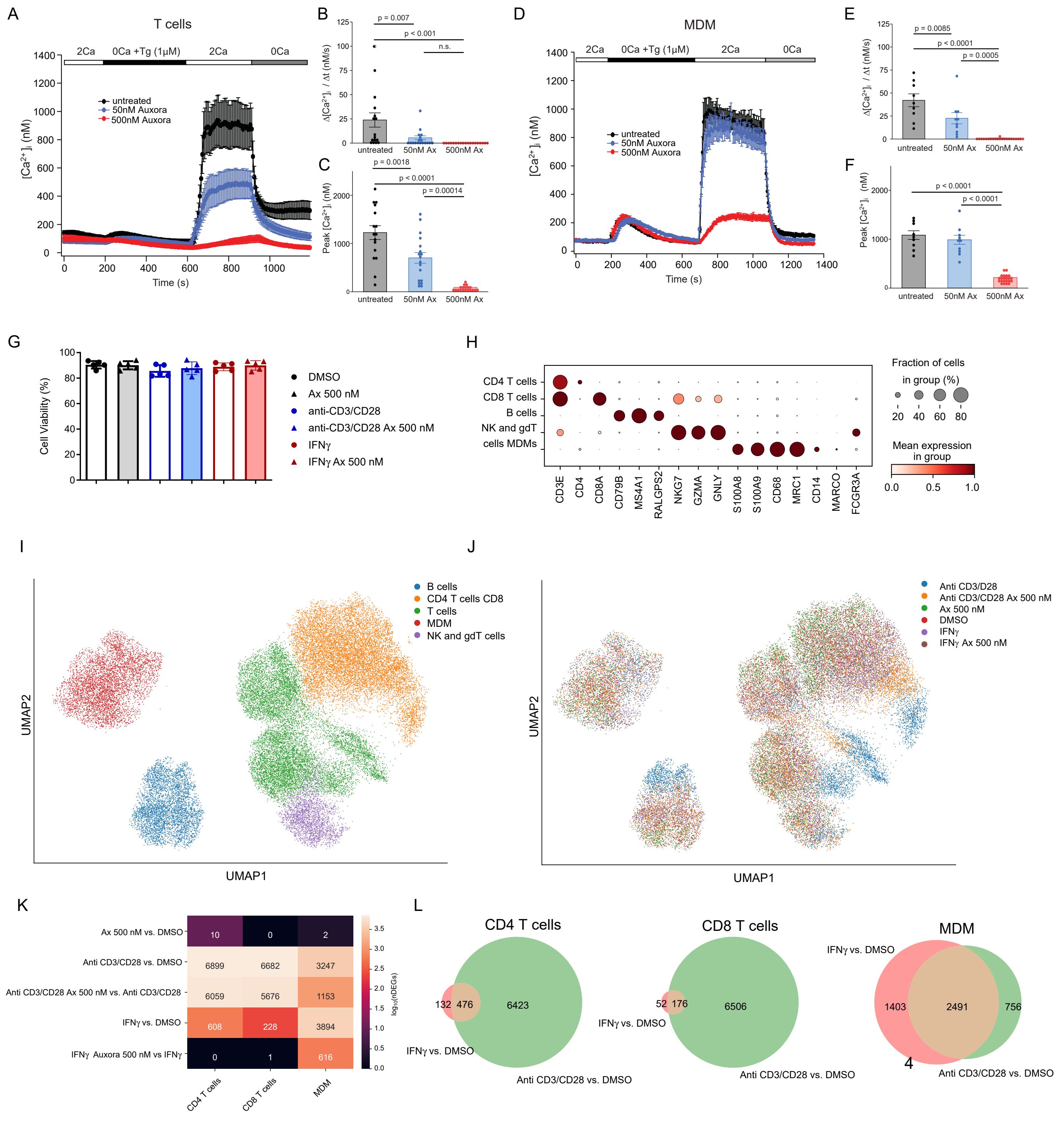

	CD4 T cells	CD8 T cells	MDM
Ax 500 nM vs. DMSO	10	0	2
Anti CD3/CD28 vs. DMSO	6899	6682	3247
Anti CD3/CD28 Ax 500 nM vs. Anti CD3/CD28	6059	5676	1153
IFNγ vs. DMSO	608	228	3894
IFNγ Auxora 500 nM vs IFNγ	0	1	616

<u>Supplemental Figure 2:</u> Auxora reverses gene transcriptional changes in PBMCs stimulated with anti-CD3/CD28 antibodies but not in PBMCs stimulated with IFN-γ. A-F, We measured store-operated calcium entry (SOCE) in Fura-2 loaded cells by administering thapsigargin (Tg, 1 μM) in Ca^{2+}-free solution followed by the addition of extracellular Ca^{2+} (2 mM) containing media. **A**, Time course of calcium measures in peripheral T cells. **B**, Rate of calcium influx after addition of extracellular Ca2+ following store depletion. **C**, Total amount of calcium over baseline entering the cell in the five minutes after switch to Ca^{2+} (2 mM) containing media. **D-F**, same as **A-C** in monocyte-derived macrophages (MDM). PBMCs were isolated from 3 different healthy donors. Bar graphs represent mean ± SEM. T cells, untreated n = 16 cells; 50 nM Auxora n = 18 cells; 500 nM Auxora n = 19 cells. MDM, untreated n = 9 cells; 50 nM Auxora n = 10 cells; 500 nM Auxora n = 22 cells. P values were calculated using ANOVA. PBMCs were plated for 1 day and treated with DMSO or Auxora (Ax) 500 nM for 2 h. Then, cells were exposed to anti-CD3/CD28 antibodies, IFN-γ or media as control. scRNAseq was performed after 24 h. **G**, Cell viability measured using a Cellometer K2. **H**, Dot plot illustrates expression of marker genes used to annotate scRNA-seq data. Dot size reflects the percentage of cells in a cluster expressing each gene; dot color reflects expression level. and cells induced by the different treatments. **I** and **J**, UMAP plot showing integrated analysis of scRNA-seq data from cells derived from cultured PBMCs. N=5 subjects. **K**, Heatmap showing the number of differentially expressed genes for each comparison. **L**, Venn diagrams indicate overlap between differentially expressed genes induced by treatment with anti CD3/CD8 antibodies or recombinant IFN-γ.

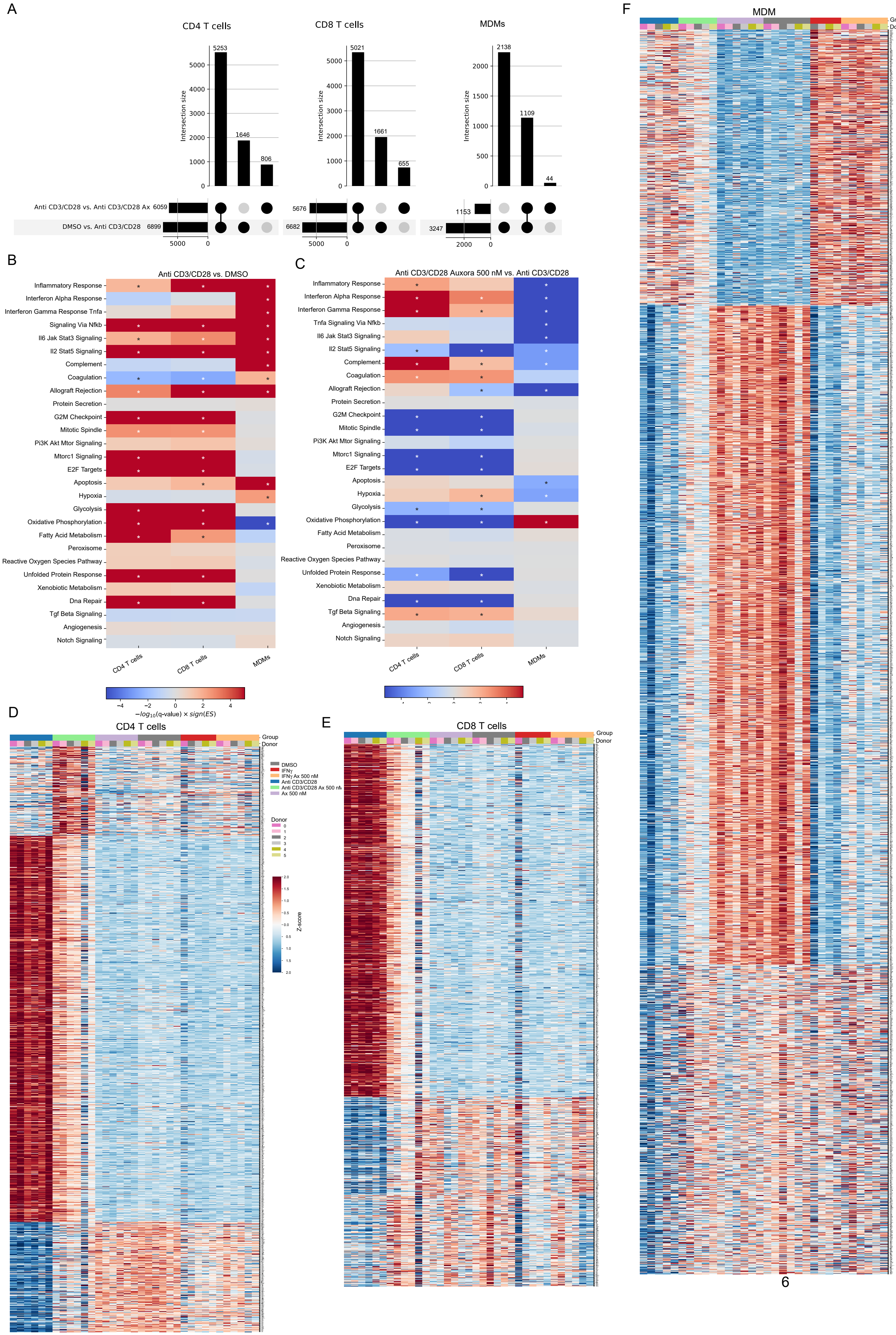
A
CD4 T cells
CD8 T cells
MDMs
Intersection size
5253
1646
806
5021
1661
655
2138
1109
44
Anti CD3/CD28 vs. Anti CD3/CD28 Ax 6059
DMSO vs. Anti CD3/CD28 6899
5676
6682
1153
3247
B
Anti CD3/CD28 vs. DMSO
C
Anti CD3/CD28 Auxora 500 nM vs. Anti CD3/CD28
Inflammatory Response
Interferon Alpha Response
Interferon Gamma Response
Tnfa Signaling Via Nfkb
Il6 Jak Stat3 Signaling
Il2 Stat5 Signaling
Complement
Coagulation
Allograft Rejection
Protein Secretion
G2M Checkpoint
Mitotic Spindle
Pi3K Akt Mtor Signaling
Mtorc1 Signaling
E2F Targets
Apoptosis
Hypoxia
Glycolysis
Oxidative Phosphorylation
Fatty Acid Metabolism
Peroxisome
Reactive Oxygen Species Pathway
Unfolded Protein Response
Xenobiotic Metabolism
Dna Repair
Tgf Beta Signaling
Angiogenesis
Notch Signaling
CD4 T cells
CD8 T cells
MDMs
−log10(q-value) × sign(ES)
D
CD4 T cells
Group
Donor
DMSO
IFNγ
IFNγ Ax 500 nM
Anti CD3/CD28
Anti CD3/CD28 Ax 500 nM
Ax 500 nM
Z-score
E
CD8 T cells
F
MDM

Supplemental Figure 3: **Auxora reverses gene transcriptional changes in PBMCs stimulated with anti-CD3/CD28 antibodies but not in PBMCs stimulated with IFN-γ.** PBMCs were plated for 1 day and treated with DMSO or Auxora (Ax) 500 nM for 2 h. Then, cells were exposed to anti-CD3/CD28 antibodies, IFN-γ or media as control. scRNAseq was performed after 24 h. **A**, Upset plots showing DEGs by T cell activation and by the effect of Auxora. **B**, Heatmap illustrating enrichment for 50 hallmark gene sets from MSigDB between anti-CD3/CD28 vs DMSO in CD4 T cells, CD8 T cells, and MDM. **C**, Heatmap illustrating enrichment for 50 hallmark gene sets from MSigDB between anti-CD3/CD28 + Auxora 500 nM vs. anti-CD3/CD28 in CD4 T cells, CD8 T cells, and MDM. Significant enrichments (q-value < 0.05) are indicated by asterisks. Heat maps of gene expression from **D**, CD4 T cells; **E**, CD8 T cells; and **F**, MDM.

		Overall
n		9
Age, median [Q1,Q3]		55.0 [50.0,64.0]
Sex at Birth, n (%)	Female	3 (33.3)
	Male	6 (66.7)
Ethnicity, n (%)	Hispanic	6 (66.7)
	Non-Hispanic	3 (33.3)
Race, n (%)	Black or African American	1 (11.1)
	Other	1 (11.1)
	Unknown or Not Reported	3 (33.3)
	White or Caucasian	4 (44.4)
BMI, median [Q1,Q3]		30.1 [27.7,35.4]
Co-enrolled in Script study? , n (%)	No	2 (22.2)
	Yes	7 (77.8)
Diabetes, n (%)	Unknown	6 (66.7)
	Yes	3 (33.3)
Hypertension, n (%)	Unknown	6 (66.7)
	Yes	3 (33.3)
Smoker, n (%)	Former smoker	1 (11.1)
	Missing or N/A	4 (44.4)
	Never smoked	4 (44.4)
Asthma, n (%)	Unknown	8 (88.9)
	Yes	1 (11.1)
Chronic obstructive pulmonary disease, n (%)	No	1 (11.1)
	Unknown	8 (88.9)
Coronary artery disease, n (%)	Unknown	9 (100.0)
Congestive heart failure, n (%)	Unknown	9 (100.0)
Azithromycin, n (%)	No	3 (33.3)
	Yes	6 (66.7)
Ongoing at randomization, n (%)	No	3 (33.3)
	None	3 (33.3)
	Yes	3 (33.3)
Remdesivir, n (%)	No	2 (22.2)
	Yes	7 (77.8)
Ongoing at randomization.3, n (%)	No	3 (33.3)
	None	2 (22.2)
	Yes	4 (44.4)
Corticosteroids, n (%)	No	2 (22.2)
	Yes	7 (77.8)
Type of Corticosteroid (choice=Dexamethasone), n (%)	Checked	7 (77.8)
	Unchecked	2 (22.2)
Ongoing at randomization.6, n (%)	None	2 (22.2)
	Yes	7 (77.8)

Supplemental Table 1: Demographic and clinical characteristics of the study population. This able presents general participant demographics, including age, sex, ethnicity, and race. Clinical variables include comorbidities, as well as medications received, and whether these were ongoing at time of study drug randomization.

Methods

Human subjects

All research involving human participants was approved by the Institutional Review Board of Northwestern University. Volunteers were enrolled in study STU00214054 and study STU00204868 at Northwestern University. We enrolled patients in a single-center, single-blind placebo-controlled ascending-dose phase 2 clinical trial to generate pharmacokinetic (PK) and pharmacodynamic (PD) data in critically ill, mechanically ventilated patients with laboratory-confirmed SARS-CoV-2 pneumonia receiving Auxora or placebo. Inclusion criteria were laboratory-confirmed SARS-CoV-2 pneumonia, moderate acute respiratory distress syndrome (PEEP > 5 cmH2O, $PaO_2/FiO_2 < 200$, and no evidence of volume overload or heart failure as the primary etiology of respiratory failure). The main exclusion criteria included mechanical ventilation > 7 days, septic shock, organ or hematologic transplant, and QTcF interval > 440 msecs. Based on our prior study(1), the primary objective was to assess the PD response of bronchoalveolar lavage (BAL) fluid T cell and monocyte and macrophage subsets and cytokine/chemokine levels to various doses of Auxora. Secondary objectives were 1) to assess safety and tolerability and 2) to determine the serum and BAL fluid PK profile of Auxora in mechanically ventilated patients with severe SARS-CoV-2 pneumonia. The trial was designed to enroll 4 patients each into three cohorts in a 3:1 drug:placebo ratio. The first cohort received Auxora or placebo once daily for 3 days, the second cohort received Auxora or placebo once daily for 4 days, and the third cohort received Auxora as a continuous infusion for 5 days. In cohorts 1 and 2, the dose of Auxora was 2.0 mg/kg (1.25 mL/kg) administered as a 4-hour infusion at 0 hour, and then 1.6 mg/kg (1 mL/kg) at 24, 48 (cohort 1), and 72 hours (cohort 2) from the start of the first infusion. In cohort 3, after an initial infusion of 2.0 mg/kg Auxora over 4 hours, the patient received continuous infusion of 1.6 mg/kg over 24 hours for 96 hours. For all cohorts, a BAL procedure was performed within 12 hours before the first dose of Auxora or placebo and 24 hours after the last dose of Auxora or placebo. One consented patient developed a prolonged QT interval prior to infusion and was excluded; another patient was recruited to fill the cohort. The trial, which coincided with the end of the COVID-19 pandemic, was stopped after only one patient who received drug was enrolled in the third cohort due to low enrollment.

Sex as a biological variable

In the present study, sex was not considered as a biological variable.

Bronchoscopy and BAL

Bronchoscopic BAL was performed as previously described(1).

SARS-CoV-2 viral titers

SARS-CoV-2 viral titers were detected with a variety of assay platforms used by our hospital's clinical laboratory, including the Cepheid Gene Expert, Abbott ID NOW, Becton-Dickinson, and a locally-developed and validated PCR in BAL fluid. Unfortunately, the specific assay used for a given sample was not included in the clinical report.

Multiplexed cytokine assay processing and analysis

We measured plasma levels of 72 cytokines, 55 of which were of sufficient quality for downstream analysis. Processing and high-level analysis were performed as previously described(2). Briefly, raw MFI values, bead counts, and standard concentrations were first stripped from the data output from Eve Technologies (Calgary, Alberta, Canada). MFI measurements with fewer than 50 bead counts were discarded. Standard curves for each cytokine were then fit for each assay run using self-starting 5-parameter logistic (5PL) models using drc 3.2-0. Cutoffs for curves with low predictive value were then determined empirically using histograms' MFI values versus standard concentrations to identify a bimodal distribution cutoff. For in-house assays, all values calculated using standard curves with MFI < 50 at 100 pg/mL were discarded. For Eve Technologies assays, all values calculated using standard curves with MFI < 50 at 10 pg/mL were discarded. Experimental values for each cytokine were then predicted using the ED function in drc with "absolute" value prediction. In rare cases where a 5PL model could not be fit for an individual cytokine assay combination, these values were excluded. Values below the lower asymptote of the model were set to a concentration of 0 pg/mL. Values above the upper asymptote were set to the value of the upper asymptote. Technical replicates (including those across assays) were collapsed by mean with NA values excluded. Analytes showing poor dynamic range were excluded from further analysis. Significance was determined using linear mixed models with a paired design that considered treatment status (before or after treatment), treatment group (Auxora or placebo), and patient and were applied for each cytokine with the formula [cytokine] ~ treated * treatment_group + (1|patient) using lme4 1.1-30. P-values were then extracted for the term "treatedTRUE:treatment_groupDrug".) BAL fluid levels of Auxora were below the limit of detection in all but 3 samples.

BAL sample processing for scRNA-seq using “no FACS” protocol

BALF samples were filtered through a 70-µm cell strainer, pelleted by centrifugation at 400 rcf for 10 min at 4°C, followed by hypotonic erythrocyte lysis using 1 ml of PharmLyse solution (BD) for 2 min, followed by another wash with 10 ml of 0.5% BSA in PBS and centrifugation. Cell pellet was resuspended in 0.5% BSA in PBS at the final concentration 1000 cells/ul. Cell concentration and viability were determined using K2 Cellometer (Nexcelom) with AO/PI reagent and cells were loaded on 10x Genomics Chip A with Chromium Single Cell 3′ V2 gel beads and reagents (10x Genomics) aiming to capture 5,000–7,000 cells per library. Libraries were prepared according to the manufacturer’s protocol (10x Genomics, CG000052_RevB). BAL sample processing for scRNA-seq using “FACS” protocol: BAL fluid samples were filtered through a 70-µm cell strainer, pelleted by centrifugation at 400 relative centrifugal force (rcf) for 10 min at 4 °C, followed by hypotonic lysis of red blood cells with 2 ml of PharmLyse (BD Biosciences) reagent for 2 min. Lysis was stopped by adding 13 ml of MACS buffer (Miltenyi Biotech). Cells were pelleted again and resuspended in 100 µl of a 1:10 dilution of Human TruStain FcX (BioLegend) in MACS buffer and a 10-µl aliquot was taken for counting using a K2 Cellometer (Nexcelom) with Acridine orange (AO)/Propidium iodide (PI) reagent. The cell suspension volume was adjusted so the concentration of cells was always < 5×10^7 cells/ ml and the fluorophore-conjugated antibody cocktail was added in a 1:1 ratio. The following antibodies were used (antigen, clone, fluorochrome, manufacturer, catalog no., final dilution): CD4, RPA-T4, BUV395, BD, 564724, 1:40; CD19, HIB19, BUV395, BD, 740287, 1:40; CD25, 2A3, BUV737, BD, 564385, 1:20; CD56, NCAM16.2, BUV737, BD, 612766, 1:20; HLA-DR, L243, eFluor450, Thermo Fisher Scientific, 48-9952-42, 1:40; CD45, HI30, BV510, BioLegend, 304036, 1:20; CD15, HI98, BV786, BD, 563838, 1:20; CD3, SK7, PE, Thermo Fisher Scientific, 12-0036-42, 1:20; CD127, HIL-7R, PECF594, BD, 562397, 1:20; CD206, 19.2, PECy7, Thermo Fisher Scientific, 25-2069-42, 1:40; CD8, SK1, APC, BioLegend, 344721, 1:40; CD14, M5E2, APC, BioLegend, 301808, 1:40; and EpCAM, 9C4, APC,

BioLegend, 324208, 1:40. After incubation at 4 °C for 30 min, cells were washed with 5 ml of MACS buffer, pelleted by centrifugation and resuspended in 500 μl of MACS buffer with 2 μl of SYTOX Green viability dye (Thermo Fisher Scientific). Cells were sorted on a FACS Aria III SORP instrument using a 100-μm nozzle. Cells were sorted into 300 μl of 2% bovine serum albumin (BSA) in Dulbecco's phosphate-buffered saline (DPBS) and immediately after sorting pelleted by centrifugation at 400 rcf for 5 min at 4 °C, resuspended in 0.5 BSA in DPBS to 1,000 cells/ μl concentration. Concentration was confirmed using a K2 Cellometer (Nexcelom) with AO/PI reagent using the 'Immune cells low RBC' program with default settings and cells were immediately used for scRNA-seq. Cells were loaded on 10x Genomics Chip A with Chromium Single Cell 3′ V2 gel beads and reagents or 10x Genomics Chip B with Chromium Single Cell 3′ V3 gel beads and reagents (10x Genomics) aiming to capture 5,000–7,000 cells per library. Libraries were prepared according to the manufacturer's protocol (10x Genomics, CG000052_RevB or CG000183_RevB).

Analysis of the flow cytometry data was performed using FlowJo v.10.7.1. using a sequential gating strategy reported in our previous publications. A fraction of cells that was not definitively resolved by our panel was labeled 'others'. Relative cell-type abundance was calculated as a percentage of all singlets/live/CD45+ cells. Statistical methods: No statistical method was used to predetermine sample size. The experiments were not randomized. The Investigators were not blinded to allocation during experiments and outcome assessment. All statistics in the manuscript are reported as specified in the figure legends. When multiple hypothesis tests were performed, the false discovery rate (FDR) was controlled using the procedure of Benjamini and Hochberg. A significance level of 0.05 was used for all tests, unless indicated otherwise.

PBMC isolation

PBMC samples were isolated from blood of 5 healthy individuals and collected in Vacutainer EDTA tubes (generic laboratory supplier) using SepMate-50 tubes (STEMCELL Technologies) following manufacturer's protocol. Blood samples were diluted 1:1 with PBS/ 2% FBS. The diluted blood was layered on top of a 15 ml Lymphoprep™ (STEMCELL Technologies) layer in the SepMate tube. The SepMate tubes were centrifuged at 1,200g for 10 min at room temperature. After centrifuging, the top plasma layer was removed as much as possible without disturbing the PBMC layer. The cells were poured from the SepMate tube into a 50 ml conical tube. The tubes containing cells were filled up to 50 ml with cold wash buffer (PBS with 2% FBS) and mixed by inverting. The tubes were centrifuged at 300g for 10 min at room temperature. After centrifuging, the supernatant was removed without disturbing the cell pellet. After resuspending the pellet with cold wash buffer, the cells were counted using the Nexcelom K2 Cellometer C automated cell counter with AO/ PI reagent. The tubes were again centrifuged at 300g for 10 min with brake set to 5 at room temperature. The supernatant was removed without disturbing the cell pellet. Aliquots containing 1.5 x 10^6 PBMCs in 200 μL Bambanker Cell Freezing Media were stored into a −80 °C freezer.

Preparation of Auxora for experimental use

The Orai1 inhibitor compound is a dry powder manufactured and provided by CalciMedica, Inc, La Jolla, CA. Auxora was dissolved in DMSO preparing a 100 mM stock solution and was used in 50 and 500 nM working concentration on cell cultures.

PBMC stimulation

PBMCs (1 x 10^5 cells/ well) were plated in RPMI (ATCC) with 10% heat-inactivated FBS with 100 U/ml Penicillin/Streptomycin in Ultra-Low Cluster 96 well plates (Costar) for 1 day. Then, PBMCs were treated with DMSO or Auxora at 50 nM or 500 nM for 2 h and T cells were exposed to ImmunoCult™ Human CD3/CD28/CD2 T Cell Activator (25 µl/ml, STEMCELL Technologies), IFNγ (50 ng/mL) or media as control. scRNAseq was performed after 24 h.

Differentiation of human monocytes derived macrophages (MDM)

Monocytes were isolated from PBMCs using the EasySep™ Human Monocyte Isolation Kit (STEMCELL Technologies). Briefly, this kit is designed to isolate $CD14^+$ $CD16^-$ monocytes from frozen PBMCs samples by negative selection of unwanted cells and platelets with antibody complexes and magnetic particles. Then, these monocytes were differentiated to MDMs using ImmunoCult™-SF Macrophage Medium plus 5 µg/mL human recombinant M-CSF (both from STEMCELL Technologies) for 4 days and 10 ng/mL LPS and 50 ng/mL IFN-γ (STEMCELL Technologies) for additional 2 days.

PBMC single-cell RNA sequencing

PBMCs single-cell suspensions were prepared as described earlier. Cells were counted using a Cellometer K2 with nucleic acid binding dyes AO to calculate total number of nucleated cells and PI to count dead cells; cell viability exceeded 85%. All manipulations were performed using wide-bore tips (Axygen; Corning). Single-cell 3′ RNA sequencing libraries were prepared using Chromium Single Cell v2 Reagent Kit and Controller (10X Genomics, Pleasanton, CA, USA). Libraries were assessed for quality (TapeStation 4200; Agilent, Santa Clara, CA, USA) and then sequenced on an HiSeq 4000 instrument (Illumina, San Diego, CA, USA). Data was processed as shown above.

Single-cell RNA-seq analysis

PBMC data were processed using Cell Ranger 1.1.0 (10x Genomics) and reads were mapped to the GRCh38 reference genome (version refdata-gex-GRCh38-2020-A, 10x Genomics). Per-sample doublet detection was performed with Scrublet(3). Data were processed using Scanpy 1.9.8, and multisample integration was performed with scvi-tools 1.1.1. An initial scVI model was constructed on 2000 HVGs with the hyperparameters n_layers = 2, n_hidden = 256, dropout_rate = 0.2, and n_latent = 10, and was trained with max_epochs = 400 and early_stopping = True. Default hyperparameters and settings were used otherwise. Leiden clustering was performed on the integrated object, and clusters characterized by low number of detected genes and transcripts, high percentage of mitochondrial genes, or co-expression of lineage-specific markers from distinct cell types (indicating doublets) were excluded. The remaining cells were re-integrated with scVI using the same hyperparameters on 1000 HVGs, with T cell receptor genes (gene symbols starting with TR) excluded prior to HVG selection to prevent clonotype-specific expression from driving the embedding of T cell populations. Leiden clustering was performed on the second integration with a resolution of 1. Cell types were identified by marker genes, computed using the sc.tl.rank_genes_groups function with default settings. Donor identity for each cell was assigned using Souporcell(4) with k = 5 expected genotype clusters per sequencing library, and cells classified as doublets or unassigned were excluded from downstream analyses.

BAL data were processed using Cell Ranger 3.1.0 (10x Genomics) and reads were mapped to the GRCh38.84 reference genome (10x Genomics Cell Ranger Human 1.2.0 GRCh38 reference).

Data was processed using Scanpy 1.10.4, and multisample integration was performed with scvi-tools 1.3.0. The scVI model was constructed on 2000 HVGs with the hyperparameters n_layers = 2, dropout_rate = 0.2, and n_latent = 10, and were trained using the settings max_epochs = 400, check_val_every_n_epoch = 2, and early_stopping = True. Default hyperparameters and settings were used otherwise. An initial round of Leiden clustering using the function sc.tl.leiden was performed on the integrated BAL object with a resolution of 1. Clusters characterized by low number of detected genes and transcripts and high percentage of mitochondrial genes were removed. Clusters containing doublets were identified as clusters simultaneously expressing lineage-specific marker genes (for example, C1QA for macrophages and CD3G for T cells) and excluded. Cell types were identified by marker genes, computed using the sc.tl.rank_genes_groups function with default settings.

For more details, see code. Differential abundance analysis was performed on fractions of identified cell clusters in each PBMC/BAL sample out of the whole sample, and the comparison was done with Mann-Whitney U tests.

Pseudobulk gene expression profiles

For several analyses of gene expression, including MOFA, PCA, differential gene expression analysis, and gene set variation analysis, we summarized gene expression for each sample and each annotated cell type from our scRNA-seq data. We required at least 50 cells to create a pseudobulk sample for a given sample and cell type. We summed integer counts for all cells across all genes, and saved this matrix for all cell types. We excluded several gene categories from the pseudobulk count matrices: mitochondrial genes, SARS-CoV-2 viral transcripts, ribosomal protein-coding genes (RPL and RPS families), and unannotated transcripts lacking canonical HGNC symbols (i.e., genes identified only by Ensembl or NCBI/RefSeq accession identifiers). These categories were excluded a priori because they reflect technical artifacts, viral rather than host signal, or transcripts without interpretable biological annotation, as we sought to elucidate established biological pathways as well as credential protein based biomarkers.

Differential gene expression analysis

To identify genes that are expressed differently between groups of samples, we used a modified pseudobulk method. This method demonstrates better consistency with the bulk approach in benchmarking studies(5), compared to single cell based methods for testing of differentially expressed genes. Additionally, it avoids the issue of inflated p-values. We used pseudobulk gene count matrices as described above and constructed a DESeq2 object using either all BAL samples or PBMC samples. For the DESeq2 model for the BAL analysis, we used ~ group + group:pair_id + group:timepoint, which models paired pre- and post-treatment BAL samples within each treatment group and tests whether the timepoint response differs between Auxora and Placebo, and we used fitType local. Differentially expressed genes were obtained from the Auxora time point 2 vs. Placebo time point 2 contrast (Wald test, $\alpha = 0.05$). For the DESeq2 model for the PBMC analysis, we used ~ pair_id + group, where pair_id controls for baseline inter-donor variability and group encodes the stimulation/Auxora condition, and we used fitType local. Differentially expressed genes were obtained from the Ax 500 nM vs. DMSO, Anti CD3/CD28 vs. DMSO, Anti CD3/CD28 Ax 500 nM vs. Anti CD3/CD28, IFNy vs. DMSO, and IFNy Auxora 500 nM vs IFNy contrasts (Wald test, $\alpha = 0.05$).

Wide-field fura-2 Ca^{+2} imaging

T cells or MDMs incubated on poly-d-lysine–coated glass-bottom dishes were loaded with Fura-2 by incubating cells in 2 μM Fura-2-AM (Invitrogen, F1221) in growth medium for 30 min at 37°C. Fura-2–containing medium was washed off, and cells were incubated for an additional 10 min before imaging. All experiments were performed at room temperature. Single-cell $[Ca^{2+}]_i$ measurements were performed as described previously(6, 7). Image acquisition and analysis were performed using SlideBook (Denver, CO). Dishes were mounted on the stage on Olympus IX71 inverted microscope, and images were acquired every 6 s at excitation wavelengths of 340 and 380 nm and an emission wavelength of 510 nm. For data analysis, regions of interest (ROIs) were drawn around single cells, background was subtracted, and F340/F380 ratios were calculated for each time point.

$[Ca^{2+}]i$ was estimated from F340/F380 ratio using the standard equation: $[Ca^{2+}]i = \beta Kd (R - Rmin)/(Rmax - R)$, where R is the F340/F380 fluorescence ratio and values of Rmin and Rmax were determined from an in vitro calibration of Fura-2 pentapotassium salt. β was determined from the F340/F380 ratio at 380 nm, and Kd is the apparent dissociation constant of Fura-2 binding to Ca^{2+} (135 nM). For each cell, the rate of SOCE ($\Delta[Ca^{2+}]i/\Delta t$) was calculated from the slope of a line fitted to three points (18 s) after the readdition of 2 mM Ca2+o. Baseline $[Ca^{2+}]_i$ was calculated by averaging $[Ca^{2+}]_i$ values over 2-min baseline for each experiment. Peak Ca^{2+} was calculated by determining the maximum Ca^{2+} concentration when 2 Ca was added back after store depletion with thapsigargin (Tg). The standard Ringer's solution used for these experiments contained the following: 155 mM NaCl, 4.5 mM KCl, 10 mM d-glucose, 5 mM Hepes, 1 mM $MgCl_2$, and 2 mM $CaCl_2$. The Ca^{2+}-free Ringer's solution was similar to the above solution except that it contained 3 mM $MgCl_2$ and 1 mM EGTA (Sigma-Aldrich) with no added $CaCl_2$. pH was adjusted to 7.4 with 1 M NaOH. The stock solution of Tg was dissolved in dimethyl sulfoxide (DMSO) and used at the indicated concentration.

Figure and manuscript preparation

Plotting was performed in Python using matplotlib and seaborn. Specific details are below and in the included code. Figures were assembled in Adobe Illustrator.

Statistics

All statistics in the manuscript are reported as specified in the figure legends. When multiple hypothesis tests were performed, the false discovery rate (FDR) was controlled using the procedure of Benjamini and Hochberg. A significance level of 0.05 was used for all tests, unless indicated otherwise.

Study approval

All research involving human participants was approved by the Institutional Review Board of Northwestern University. Volunteers were enrolled in study STU00214054 and study STU00204868 at Northwestern University. Written informed consent was obtained for each participant.

Data availability

Single-cell RNA-seq counts tables and integrated objects are available through the Gene Expression Omnibus with accession number GSE330843. Supporting data values are include in a separated excel spreadsheet.

Code availability

All code used for analysis can be found at https://github.com/NUPulmonary/Guggilla_Matsuda_Auxora_2026. ScRNA-seq data can be explored via UCSC data browsers at https://sqlifts.fsm.northwestern.edu/internal/auxora/?ds=pbmc_integrated_0424 and at https://sqlifts.fsm.northwestern.edu/internal/auxora/?ds=2025-09-30_Auxora_Human.

References

1. Grant RA, Morales-Nebreda L, Markov NS, Swaminathan S, Querrey M, Guzman ER, et al. Circuits between infected macrophages and T cells in SARS-CoV-2 pneumonia. *Nature.* 2021;590(7847):635-41.
2. Grant RA, Poor TA, Sichizya L, Diaz E, Bailey JI, Soni S, et al. Prolonged exposure to lung-derived cytokines is associated with activation of microglia in patients with COVID-19. *JCI Insight.* 2024;9(8).
3. Wolock SL, Lopez R, and Klein AM. Scrublet: Computational Identification of Cell Doublets in Single-Cell Transcriptomic Data. *Cell Syst.* 2019;8(4):281-91 e9.
4. Heaton H, Talman AM, Knights A, Imaz M, Gaffney DJ, Durbin R, et al. Souporcell: robust clustering of single-cell RNA-seq data by genotype without reference genotypes. *Nat Methods.* 2020;17(6):615-20.
5. Squair JW, Gautier M, Kathe C, Anderson MA, James ND, Hutson TH, et al. Confronting false discoveries in single-cell differential expression. *Nat Commun.* 2021;12(1):5692.
6. Feske S, Gwack Y, Prakriya M, Srikanth S, Puppel SH, Tanasa B, et al. A mutation in Orai1 causes immune deficiency by abrogating CRAC channel function. *Nature.* 2006;441(7090):179-85.
7. Soberanes S, Misharin AV, Jairaman A, Morales-Nebreda L, McQuattie-Pimentel AC, Cho T, et al. Metformin Targets Mitochondrial Electron Transport to Reduce Air-Pollution-Induced Thrombosis. *Cell Metab.* 2019;29(2):503.

Conflict of interest

Benjamin Singer holds United States Patent No. US 10,905,706 B2, Compositions and Methods to Accelerate Resolution of Acute Lung Inflammation, and serves on the Scientific Advisory Board of Zoe Biosciences. Alexander Misharin received research grants from AbbVie and Merck, and consulting fees from Boehringer Ingelheim. Sudarshan Hebbar and Kenneth Stauderman are employees of CalciMedica and own shares in CalciMedica. Richard G. Wunderink serves as consultant for CalciMedica.

Acknowledgments

This work is the result of NIH funding, in whole or in part, and is subject to the NIH Public Access Policy. Through acceptance of this federal funding, the NIH has been given a right to make the work publicly available in PubMed Central.

C.A.G. is supported by NIH/NHLBI K23HL169815, a Parker B. Francis Opportunity Award, and an American Thoracic Society Unrestricted Grant. R.G.W. is supported by NIH/NIAID U19 AI135964, R01 AI158530; NHLBI R01 HL149883, P01 HL154998; U01TR003528. B.D.S. is supported by NIH awards R01HL149883, R01HL153122, P01HL154998, P01AG049665, U19AI135964, and U19AI181102. G.R.S.B. is supported by the NIH (U19AI135964, P01AG049665, R01HL147575, P01HL071643, U54AG079754, R01HL145478, R01HL147290, R01HL173940, U19AI181102, and R01HL154686); the US Department of Veterans Affairs (I01CX001777); Simpson Querrey Lung Institute for Translational Sciences. A.V.M. was supported by the NIH (grant nos. U19AI135964, P01AG049665, P01HL154998, P01HL169188, U19AI181102, R01HL153312, R01HL158139, and R01ES034350), and research grants from AbbVie and Merck.

We thank all patients and volunteers who provided their samples and data for this study and their families. This research was supported in part through a generous gift from Kimberly Querrey and Louis A. Simpson. This research was supported by the Simpson Querrey Lung Institute for Translational Science (SQLIFTS), Northwestern University. This research was also supported by the computational resources and staff contributions provided for the Quest high-performance computing facility at Northwestern University, which is jointly supported by the Office of the Provost, the Office for Research and Northwestern University Information Technology. This research was also supported in part through the computational resources and staff contributions provided by the Genomics Compute Cluster, which is jointly supported by the Feinberg School of Medicine, the Center for Genetic Medicine and Feinberg's Department of Biochemistry and Molecular Genetics, the Office of the Provost, the Office for Research and Northwestern Information Technology. The Genomics Compute Cluster is part of Quest, Northwestern University's high-performance computing facility, with the purpose of advancing research in genomics. Northwestern University Flow Cytometry Core Facility is supported by the National Cancer Institute Cancer Center support grant (no. P30 CA060553) awarded to the Robert H. Lurie Comprehensive Cancer Center. Sorting was performed on a BD FACS Aria SORP cell sorter purchased with the support of the National Institutes of Health (NIH, grant no. 1S10OD011996-01). Integrative genomic services were performed by the Metabolomics Core Facility at Robert H. Lurie Comprehensive Cancer Center of Northwestern University.
See Supplemental Acknowledgments for consortium details.

Author contributions

Conceptualization: S.M.C.M., R.G.W., B.D.S., G.R.S.B., and A.V.M. Methodology: S.M.C.M., V.G., C.A.G., R.G.W., and A.V.M. Software: S.M.C.M., V.G., C.A.G., L.C., S.W.F., M.S. and A.V.M. Validation: S.M.C.M., V.G., C.A.G., R.G.W., and A.V.M. Formal analysis: S.M.C.M, V.G., C.A.G., L.C., S.W.F., R.G.W., and A.V.M. Investigation: S.M.C.M., C.A.G., K.D., Z.Y., Z.L., R.A.G., H.K.D., R.G.W., and A.V.M. Resources: S.M.C.M., V.G., C.A.G., K.D., Z.Y., Z.L., S.S., R.A.G., M.P., S.H., K.S., H.K.D., C.I.P., L.M.-N., R.G.W., B.D.S., G.R.S.B. and A.V.M. Data curation: S.M.C.M, V.G., C.A.G., L.C., Z.Y., Z.L., R.G.W., B.D.S., G.R.S.B. and A.V.M. Writing – all aspects: S.M.C.M., R.G.W., B.D.S., G.R.S.B., and A.V.M. Writing – review & editing: S.M.C.M. Visualization: S.M.C.M. and V.G. Supervision: S.M.C.M., R.G.W., G.R.S.B., B.D.S., and A.V.M. Project administration: S.M.C.M., R.G.W., B.D.S., G.R.S.B., and A.V.M. Funding acquisition: R.G.W., B.D.S. G.R.S.B., and A.V.M. S.M.C.M and V.G authorship order was determined based on who took the lead in drafting the manuscript and conducting the primary in vitro experiments.

NU Script study

First name	Last name	Affiliation(s) at Northwestern University
Aishvarya	Agrawal	Division of Pulmonary and Critical Care Medicine
Alan	Hauser	Department of Microbiology-Immunology
Alec	Peltekian	McCormick School of Engineering
Alexander	Misharin	Division of Pulmonary and Critical Care Medicine
Alexis	Wolfe	Division of Pulmonary and Critical Care Medicine
Alison	Szabo	Division of Pulmonary and Critical Care Medicine
Alok	Choudhary	Department of Electrical and Computer Engineering; Department of Computer Science
Alvaro	Donayre	Division of Pulmonary and Critical Care Medicine
Anahid	Moghadam	Department of Civil and Environmental Engineering
Anjana	Yeldandi	Department of Pathology
Ankit	Agrawal	Department of Electrical and Computer Engineering
Ankit	Bharat	Division of Thoracic Surgery; Division of Pulmonary and Critical Care Medicine
Anna	Pawlowski	Northwestern Medicine Enterprise Data Warehouse
Anthony	Joudi	Division of Pulmonary and Critical Care Medicine
Arjun	Tambe	Division of Pulmonary and Critical Care Medicine
Ashley	Smith-Nunez	Division of Pulmonary and Critical Care Medicine
Benjamin	Singer	Division of Pulmonary and Critical Care Medicine; Department of Biochemistry and Molecular Genetics
Benjamin	Ulrich	Division of Pulmonary and Critical Care Medicine
Betty	Tran	Division of Pulmonary and Critical Care Medicine
Bharat	Balan	Division of Pulmonary and Critical Care Medicine
Brian	White	Division of Pulmonary and Critical Care Medicine
Cara	Gottardi	Division of Pulmonary and Critical Care Medicine
Catherine	Gao	Division of Pulmonary and Critical Care Medicine
Chao	Qi	Department of Pathology
Chiagozie	Pickens	Division of Pulmonary and Critical Care Medicine
Clara	Schroedl	Division of Pulmonary and Critical Care Medicine
Daniel	Amusin	Department of Microbiology-Immunology, Division of Infectious Disease, Feinberg School of Medicine
Daniel	Meza	Division of Pulmonary and Critical Care Medicine
Daniel	Schneider	Northwestern Medicine Enterprise Data Warehouse
David	Lee	Division of Pulmonary and Critical Care Medicine
Dulce	Garcia	Division of Infectious Disease, Feinberg School of Medicine
Egon	Ozer	Department of Microbiology-Immunology
Elen	Gusman	Division of Pulmonary and Critical Care Medicine
Elisheva	Shanes	Department of Pathology
Emily	Leibenguth	Division of Pulmonary and Critical Care Medicine
Emily	Olson	Division of Pulmonary and Critical Care Medicine, Feinberg School of Medicine

Erica	Hartmann	Department of Civil & Environmental Engineering
Erin	Korth	Division of Pulmonary and Critical Care Medicine
Estefani	Diaz	Robert H. Lurie Comprehensive Cancer Research Center
Francisco	Martinez	Division of Pulmonary and Critical Care Medicine
Gabrielle	Matias	Division of Pulmonary and Critical Care Medicine
Scott	Budinger	Division of Pulmonary and Critical Care Medicine
Helen	Donnelly	Division of Pulmonary and Critical Care Medicine
Hiam	Abdala-Valencia	Division of Pulmonary and Critical Care Medicine
Jack	Sumner	Department of Civil & Environmental Engineering
Jacob	Sznajder	Division of Pulmonary and Critical Care Medicine
Jacqueline	Kruser	
Jakub	Glowala	Division of Pulmonary and Critical Care Medicine
James	Walter	Division of Pulmonary and Critical Care Medicine
Jamie	Rowell	Feinberg School of Medicine
Jason	Arnold	Department of Medicine
Jessica	Blank	Division of Pulmonary and Critical Care Medicine
John	Coleman	Division of Pulmonary and Critical Care Medicine
Jon	Lomasney	Department of Pathology; Department of Pharmacology
Jordyn	Deveney	Division of Pulmonary and Critical Care Medicine, Feinberg School of Medicine
Joseph	Bailey	Division of Pulmonary and Critical Care Medicine
Judd	Hultquist	Division of Infectious Disease
Justin	Fiala	Division of Pulmonary and Critical Care Medicine
Justin	Starren	Division of Health and Biomedical Informatics, Department of Preventive Medicine
Karen	Ridge	Division of Pulmonary and Critical Care Medicine
Karolina	Senkow	Division of Pulmonary and Critical Care Medicine
Katy	Bond	Department of Civil and Environmental Engineering, Interdisciplinary Biological Sciences (IBiS) Program, SynBAS NRT
Katie	Helmin	Division of Pulmonary and Critical Care Medicine
Kevin	Grudzinski	Division of Pulmonary and Critical Care Medicine
Khalilah	Gates	Division of Pulmonary and Critical Care Medicine
Lacy	Simmons	Division of Infectious Disease
Lars	Johnson	Division of Pulmonary and Critical Care Medicine
Lesley	Pinzon	Division of Pulmonary and Critical Care Medicine, Feinberg School of Medicine
Lindsey	Gradone	Division of Pulmonary and Critical Care Medicine
Lisa	Wolfe	Division of Pulmonary and Critical Care Medicine
Lucy	Luo	Medical Scientist Training Program
Luisa	Morales-Nebreda	Division of Pulmonary and Critical Care Medicine
Luisa	Cusick	Division of Pulmonary and Critical Care Medicine

Luke	Rasmussen	Department of Preventive Medicine
Manu	Jain	Division of Pulmonary and Critical Care Medicine
Marc	Sala	Division of Pulmonary and Critical Care Medicine
Maxwell	Schleck	Division of Pulmonary and Critical Care Medicine
Melissa	Ross	Division of Pulmonary and Critical Care Medicine
Melissa	Querrey	Division of Thoracic Surgery; Division of Pulmonary and Critical Care Medicine
Mengjia	Kang	Division of Pulmonary and Critical Care Medicine
Michael	Cuttica	Division of Pulmonary and Critical Care Medicine
Michelle	Prickett	Division of Pulmonary and Critical Care Medicine
Nandita	Nadig	Division of Pulmonary and Critical Care Medicine
Nathaniel	Rhodes	Midwestern University
Navdeep	Chandel	Division of Pulmonary and Critical Care Medicine
Nikolay	Markov	Division of Pulmonary and Critical Care Medicine
Paige	Messa	Division of Pulmonary and Critical Care Medicine, Feinberg School of Medicine
Peter	Sporn	Division of Pulmonary and Critical Care Medicine; Medical Service, Jesse Brown Veterans Affairs Medical Center
Prasanth	Nannapaneni	Northwestern Medicine Enterprise Data Warehouse
Iris	Liu	Division of Pulmonary and Critical Care Medicine
Rachel	Kadar	Critical Care Medicine, Anesthesiology
Ramon	Lorenzo-Redondo	Division of Infectious Disease
Ravi	Kalhan	Division of Pulmonary and Critical Care Medicine
Rebecca	Clepp	Division of Pulmonary and Critical Care Medicine
Richard	Wunderink	Division of Pulmonary and Critical Care Medicine
Richard	Morimoto	Department of Molecular Biosciences
Rogan	Grant	Division of Pulmonary and Critical Care Medicine
Ruben	Mylvaganam	Division of Pulmonary and Critical Care Medicine
Samuel	Fenske	Division of Pulmonary and Critical Care Medicine
Scott	Laurenzo	Division of Pulmonary and Critical Care Medicine
Sean	Smith	Division of Pulmonary and Critical Care Medicine
SeungHye	Han	Division of Pulmonary and Critical Care Medicine
Sophia	Nozick	Division of Microbiology-Immunology
Srinivas	Panchamukhi	Division of Pulmonary and Critical Care Medicine
Stephanie	Eisenbarth	Division of Allergy and Immunology
Suchitra	Swaminathan	Division of Rheumatology; Lurie Cancer Center; Feinberg School of Medicine
Susan	Russell	Division of Pulmonary and Critical Care Medicine
Taylor	Poor	Division of Pulmonary and Critical Care Medicine
Thaddeus	Cybulski	Division of Pulmonary and Critical Care Medicine
Theresa	Lombardo	Division of Pulmonary and Critical Care Medicine

Theresa	Walunas	Medicine (General Internal Medicine), Medical Social Sciences, Microbiology-Immunology, Preventive Medicine (Biostatistics and Informatics)
Thomas	Stoeger	Department of Chemical and Biological Engineering, McCormick School of Engineering
Timothy	Rowe	Division of Pulmonary and Critical Care Medicine
Vijeeth	Guggilla	Feinberg School of Medicine
Wan-Ting	Liao	Feinberg School of Medicine
Yuan	Luo	Department of Preventive Medicine; McCormick School of Engineering
Yuliana	Sokolenko	Division of Pulmonary and Critical Care Medicine
Zhan	Yu	Division of Pulmonary and Critical Care Medicine
Ziyan	Lu	Division of Pulmonary and Critical Care Medicine
